# Using attendance data for social network analysis of a community-engaged research partnership

**DOI:** 10.1017/cts.2020.571

**Published:** 2020-12-21

**Authors:** Kimberly S. Vasquez, Shirshendu Chatterjee, Chamanara Khalida, Dena Moftah, Brianna D’Orazio, Andrea Leinberger-Jabari, Jonathan N. Tobin, Rhonda G. Kost

**Affiliations:** 1 Community and Collaboration Core, The Rockefeller University, Center for Clinical and Translational Science, New York, NY, USA; 2 Department of Mathematics, City University of New York, City College & Graduate Center, New York, NY, USA; 3 Center for Excellence for Practice-Based Research and Learning, Clinical Directors Network (CDN), New York, NY, USA; 4 Public Health Research Center, New York University Abu Dhabi, Abu Dhabi, United Arab Emirates

**Keywords:** Interdisciplinary, community-based participatory research, community engagement, partnership development, collaboration outcomes, social network analysis

## Abstract

**Background:**

The Rockefeller University Center for Clinical and Translational Science (RU-CCTS) and Clinical Directors Network (CDN), a Practice-Based Research Network (PBRN), fostered a community–academic research partnership involving Community Health Center (CHCs) clinicians, laboratory scientists, clinical researchers, community, and patient partners. From 2011 to 2018, the partnership designed and completed Community-Associated Methicillin-Resistant *Staphylococcus Aureus* Project (CAMP1), an observational study funded by the National Center for Advancing Translational Sciences (NCATS), and CAMP2, a Comparative Effectiveness Research Study funded by the Patient-Centered Outcomes Research Institute (PCORI). We conducted a social network analysis (SNA) to characterize this Community-Engaged Research (CEnR) partnership.

**Methods:**

Projects incorporated principles of Community-Based Participatory Research (CAMP1/2) and PCORI engagement rubrics (CAMP2). Meetings were designed to be highly interactive, facilitate co-learning, share governance, and incentivize ongoing engagement. Meeting attendance formed the raw dataset enriched by stakeholder roles and affiliations. We used SNA software (Gephi) to form networks for four project periods, characterize network attributes (density, degree, centrality, vulnerability), and create sociograms. Polynomial regression models were used to study stakeholder interactions.

**Results:**

Forty-seven progress meetings engaged 141 stakeholders, fulfilling 7 roles, and affiliated with 28 organizations (6 types). Network size, density, and interactions across organizations increased over time. Interactions between Community Members or Recruiters/Community Health Workers and almost every other role increased significantly across CAMP2 (*P* < 0.005); Community Members’ centrality to the network increased over time.

**Conclusions:**

In a partnership with a highly interactive meeting model, SNA using operational attendance data afforded a view of stakeholder interactions that realized the engagement goals of the partnership.

## Introduction

The development of effective community-engaged translational science teams is a priority for the National Center for Advancing Translational Sciences (NCATS) Clinical and Translational Science Awards (CTSA) program. Successful community–academic partnerships can better focus research priorities, improve study design, implementation, and dissemination, and ultimately improve population health [1–5].

Community-Based Participatory Research (CBPR) is a well-established model for fostering academic–community partnerships [6–8]. In CBPR, partners share authority and responsibility, and undertake respectful negotiation throughout the conceptualization, development, and conduct of the research to ensure that the concerns, interests, and needs of each party are addressed [9]. Extensive scholarly analysis of CBPR has delineated a robust logic model, psychometric constructs supporting the development of partnership, correlates for developing trust, and measures of associated social capital and health outcomes impact [5,7]. Functional models have been proposed to help research teams to operationalize CBPR, including Community Engagement Studios [10], Community Engagement Components Practical Model [11], Community Engagement Framework from the NYC Department of Health and Mental Hygiene [12], Community-Engaged Research Navigation (CEnR-Nav) [13], and others. A major sponsor of CBPR, the Patient-Centered Outcomes Research Institute (PCORI) requires grantees to incorporate specific principles and practices to assure CBPR [14,15].

Scales and measures, some reliable and valid, have been proposed for assessing specific aspects of a partnership [5,16,17]. Most require collaborating stakeholders to complete serial assessments of their partnership experiences. To date, no set of measures has been adopted as a gold standard to evaluate collaboration in community-engaged translational research at CTSAs. The evaluation of CEnR partnerships remains challenging.

From 2010 to 2018, The Rockefeller University Center for Clinical and Translational Science (RU-CCTS) and Clinical Directors Network (CDN), a Practice-Based Research Network (PBRN), fostered a multi-stakeholder CEnR partnership that developed, conducted, and completed two extramurally funded clinical translational research projects, Community-Associated Methicillin-Resistant *Staphylococcus Aureus* Projects, CAMP1 and CAMP2, each addressing aspects of community-acquired treatment-resistant infections in Federally Qualified Health Centers (FQHCs).

CAMP1 was developed with a CTSA-funded CEnR Pilot Award (2010–2011) to build capacity and foster the partnership using a CBPR-inspired model to engage basic scientists and communities, CEnR-Nav model [13]. Community clinicians, basic scientists, clinician-scientists, PBRN, and Community Engagement (CE) core staff explored unmet clinical needs among patients attending FQHCs, and research priorities for community clinicians and basic scientists. The partners assembled prep-to-research data to align aims, refine feasibility, and successfully compete for extramural funding. Grant support was awarded through a CTSA Administrative Supplement (NIH/NCATS 8 UL1 TR000043) that supported the implementation of CAMP1 from 2011 to 2015. CAMP1 built CEnR research infrastructure for full spectrum translational research among the community–academic partners, including six NYC area FQHCs serving predominantly minority and underserved populations. CAMP1 findings formed the preliminary data for the Comparative Effectiveness Research (CER) trial CAMP2. CAMP2 Development meetings engaged an expanded group of stakeholders, incorporating PCORI principles in its design, resulting in a successful funding award (PCORI/CER-1402-10800) to support CAMP2 implementation.

By multiple objective measures, the CAMP partnership was successful: CAMP1/2 enrolled more than 270 participants, accomplished its scientific and patient-centered aims, and produced a range of publications spanning the phases of translational research [18] that describe the basic biology and clinical aspects of CA-MRSA in the study population [19–23], the outcome of the CER intervention [24,25] features of the CEnR team science model [13], and disseminated results to diverse audiences [24–26]. Qualitative measures of collaboration were not collected prospectively in the CAMP1/2 projects. In retrospect, given the success of the CAMP projects, we sought another means to characterize how the research partnership grew and was sustained using data available from project operations.

Social network analysis (SNA) is a mathematical approach used to describe, and characterize interactions among members of a group, and to provide data to visualize those relationships [27]. In addition to interactions, SNA can identify the most connected and potentially influential members within a network and illuminate the dynamics of relationships over time [28].

SNAs focused on academic–research collaborations have used different approaches to assess interactions, including using co-authorship on research studies or grant proposals to form the basis of the network [29–31]. A limitation of this approach is that it does not capture how collaborations start, or evolve over time [28]. Using this archival approach, community stakeholders or partners who were not granted co-authorship nor listed among grant key proposals are not included in the SNA. Several reported SNAs that studied research collaborations that included community-engaged partners used different sources of data to form the network. In an analysis of a state-wide health policy coalition, investigators used a cross-sectional survey of self-reported interaction data as the basis for an SNA that demonstrated expansion of the coalition over time, and formed the basis for recommendations for enhancing collaboration [27]. Another SNA evaluated a community-based cancer disparities partnership formed between a University Cancer Center and a coalition of 20 member chapters using 3 years of survey data, meeting attendance records, and meeting minutes to form the basis of SNA showing increased interaction and interdependence among chapter organizations and less dependence on the cancer center [32]. Researchers have also sought to simplify the burdens of data collection for SNA using the perspectives of a few key informants, and survey findings from representative stakeholder groups to draw conclusions about current, preferred, and projected social networks for SNA and propose approaches to improve CBPR collaborations [33].

In the absence of serial survey data, simple measures of network formation and integrity using data extracted from project operations would be a useful option for teams not able to conduct extensive qualitative analyses. We sought to test whether SNA using attendance at CAMP1/2 project meetings would offer insights into the evolution of the CEnR CAMP partnership. The aims were to (1) conduct an SNA using attendance data as the surrogate for engagement, (2) visualize the network over time and characterize typical network attributes, and interactions within the network, and (3) examine whether stakeholder engagement in the network is positively associated with study accrual and retention.

## Methods

### Ethical Review

All research protocols for CAMP1 and CAMP2 (NCT02566928) were approved by The Rockefeller University Institutional Review Board (IRB), or the IRBs of The Biomedical Research Alliance of New York (BRANY), Weill Cornell Medical Center, and CDN, directly or under reliance agreements before research began at the respective sites.

### Project Phases

The project lifespan was divided into 4 periods: *CAMP1 Development –* 3 meetings (December 2010–July 2011)*; CAMP1 Implementation –* 13 meetings (October 2011–February 2013); *CAMP2 Development* – 12 meetings (May 2013–July 2015); and *CAMP2 Implementation –* 19 meetings (July 2015–May 2018).

### Stakeholders

Any individual who attended at least one project meeting within any phase of CAMP1 or CAMP2 was considered a stakeholder for the SNA. To assemble the stakeholders for the CAMP1 CHC Clinician Advisory Committee (CAC) to the project, the PBRN leader and RU-CCTS collaboration core directors recruited leaders from NYC FQHCs, RU-CCTS basic scientists, academic clinician-scientists, CE core staff, and CDN staff for project development. The FQHC site Medical Directors nominated themselves and/or other community clinicians and health center staff as stakeholders to the CAMP1 project. The basic scientists nominated other stakeholders from their laboratories to join the project. Additional stakeholders from the community with relevant knowledge or lived experience were invited as stakeholders as the project developed.

CAMP2 stakeholders were assembled through the creation of the Clinician and Patient Stakeholder Advisory Committee (CPSAC), which included patient stakeholders from CAMP1, NYC FQHC staff from two CAMP1 sites, and four new Community Health Centers (CHC)s and emergency departments (ED)s recruited by the Principal Investigator. CDN staff, clinician-scientists, and CE core staff were included. Each CHC site nominated community clinicians and a patient representative to the CPSAC. Other stakeholders included a local barbershop owner/community health organizer who had collaborated on a prior project to raise awareness of MRSA among barbers and their clientele. CAMP2 funds were allocated to the PBRN to hire a community recruiter for each site to reduce the study burden for staff, and community health workers to inform culturally appropriate implementation [24].

### Roles and Affiliations

Stakeholders were characterized by their organizational affiliation and role in the project using the information in protocol documents. Roles and affiliations were coded independently by two authors (KV and RK) and compared. Any rating differences were reconciled through discussion with additional team members.

### Attendance

CAMP1/2 team-wide progress meetings were held every 1–2 months, in person or by teleconference. Attendance was voluntary and encouraged. Individual stakeholder attendance was recorded at every meeting.

### Engagement by Design

CAMP1 and CAMP2 embodied PCORI principles of engagement by design [15], including (1) *Reciprocal partnership* through shared resources and shared decision-making at every step of project development and implementation. In addition, CDN provided logistical and financial support for CAMP2 meetings; (2) *Co-Learning* through bidirectional capacity building, joint problem-solving, and continuing education that appealed to diverse stakeholders; (3) *Partnership* characterized by the inclusion of priority populations through recruitment in FQHCs and community hospitals, and the participation of community members, patient stakeholders from CAMP1, and patient representatives in the CAMP2 CPSAC; (4) *Transparency, honesty and trust,* characterized by ongoing multilevel communication with all stakeholders throughout the partnership to ensure transparency and built trust through shared experiences and time.

### Interactive Meetings

To operationalize engagement, meetings followed semi-structured agendas determined by the stakeholders and the current needs of the project. Development meetings entailed capacity-building with partnership members teaching each other. Community members learned about basic science and research methods, and academics learned about health center populations, health priorities, and operational realities. All attendees participated in collaborative concept generation, priority setting, protocol, and grant co-writing, study implementation planning, and operational problem-solving. Implementation meetings involved a review of study progress, strategies to increase recruitment, engagement, and retention of participants, identified barriers and opportunities, assessments, retention, operational problem-solving, analysis, and opportunities for dissemination [24]. Stakeholders were also engaged outside of the scheduled progress meetings by CDN staff and CE directors through emails or phone calls.

### Incentives

Project activities were designed to enhance the return on investment of stakeholders’ time. Activities included professional development opportunities for clinicians and staff with the option to earn continuing medical education credit; opportunities for quality improvement at the health center practices as a result of the research; the ability to develop research that matters to the health center and the community; the potential for early access to drugs, devices, procedures or informatics tools that have the potential for reducing health disparities; and recognition that the health centers, clinicians, and health center staff receive in publications and presentations. PCORI reviewed the CAMP2 project design thoroughly to make sure stakeholders were properly compensated for their time.

### Study Milestones

Study milestones for CAMP1 and CAMP2 were collected for each site: Recruitment was defined as the number of participants signing informed consent for screening. Enrollment was defined as the number of participants who pass screening and were enrolled or randomized into the hypothesis testing part of the study. Retention was defined as the number of participants completing all study visits.

### Social Network Analysis

Stakeholder attendance at progress meetings formed the basis for the SNA, with the co-attendance of two individuals defining an interaction. Key constructs for the SNA are defined, and our approach to each is described in Table 1.

**Table 1.** Social network analysis construct definition and approach

Construct definition	Approach
**Social Network Analysis (SNA)** is a mathematical approach to graph interactions. Network members can be individuals, groups, organizations, or other units. Social networks have characteristics that can be measured and tracked over time. SNA can afford insight into the dynamics of the network, and reveal successes, limitations, and opportunities. SNA can be used retrospectively to understand what already transpired within a network, and increasingly is used prospectively to evaluate a collaboration during its development to identify opportunities to strengthen the network and enhance its success [34].	Attendance at one or more CAMP1/2 progress meetings qualified an individual as a stakeholder for the SNA. The attendance of two individual stakeholders at the same meeting defined an interaction between them in the SNA. We compiled the attendance records for each CAMP1/2 project meeting to create a matrix where each row represents an individual stakeholder (present/absent) and each column represents an event (meeting date). We used SNA software (Gephi) to convert the two-mode (person→event) matrix to a one-mode (person→person) matrix [35–37]. Stakeholders’ roles in the project, and institutional and site affiliations were used to enrich the dataset.
	We performed the Kolmogorov–Smirnov test [38] to compare interactions among role types.
**Node:** Each member of the network is represented as a node in the SNA.	
**Edge:** The interaction between two nodes in a network forms an “edge,” represented as a line drawn between two nodes. A *directed edge* is an ordered pair (drawn as a line with an arrow); an *undirected edge* disregards any sense of direction and treats both nodes equally in the interaction.	When two individuals are present at the same meeting, this defines an interaction, expressed as an “edge” in the SNA. When interactions are analyzed between groups (e.g., roles or affiliations) they also form edges. All edges in the CAMP SNA are undirected.
**Edge Weight:** The weight of an edge is the sum of interactions along that edge (between the two connected nodes).	
**Node Degree: **There are two ways to compute the degree (measure of all interactions) of a given node in the networks. If one views the network as an *unweighted network*, where two nodes (stakeholders) are linked by an edge if they participated in at least one meeting, the degree of a stakeholder is the number of other stakeholders who co-attended at least one meeting. Alternatively, if one views the network as a *weighted network*, where the weight of an edge joining two nodes (stakeholders) is the total number of meetings they co-attended, then the degree of a stakeholder is the total number of interactions with other stakeholders.	We used the weighted notion of degree to assess interactions in the network, and for the analysis of degree distribution. We considered both weighted and unweighted notions of degree in the vulnerability analyses.
Degree distribution is the probability distribution of stakeholder degrees over the whole network.	We assessed degree distribution to understand whether changes in degree in the network occurred among a few or many of the stakeholders. We used Two-sample Wilcoxon tests to compare the degree distribution among stakeholders across the four different project phases.
**Centrality:** Measures of *centrality* are designed to quantify the “importance” of a vertex (node) in the network. There are three classic types of centrality measures. *Closeness centrality* measures attempt to capture how many nodes are “close” to a given node *x* within a network graph (e.g., have a small number of edges separating them). *Between-ness centrality* measures are based upon the perspective that “importance” relates to where a vertex is located with respect to the paths in the network graph. The third class of centrality measure, based on “status,” “prestige,” or “rank,” seeks to capture the idea that the more central the neighbors of a vertex are, the more central that vertex is. These measures typically can be expressed in terms of eigenvector solutions of appropriately defined linear systems of equations. There are many e*igenvector centrality* measures initially developed by Bonacich and others [39,40].	We used the “eigen_centrality” function of the R software, which is based on the method developed in Bonacich [41] as the measure of most interest because it incorporates measures of the density of the network, and of the centrality of the other nodes that are connected, in calculating a given node’s centrality score.
**Vulnerability:** Vulnerability is a measure of the susceptibility of a network to the loss of members (damage). Damage can be *random,* where all parts of the network under consideration are equally likely to receive the damage, as there is *no a priori* reason behind such damage. Alternatively, damage can be *purposeful,* where the network structure is known, and the loss is targeted to the location of the network where minimum effort gives maximum damage. For a given network, one can test a given network’s vulnerability to both random and purposeful damage.	For each network to test vulnerability to random damage, we removed one randomly chosen node at a time from the network until two or more major components of the remaining network become disconnected, and we counted the number of nodes removed. After 1000 replications, we report the average number of nodes that need to be removed to disconnect each of the networks. We compared the averages as percentages of the nodes in each study phase. To study the effect of purposeful damage, we sorted the nodes in descending order of degree, and determined how many high-degree nodes need to be removed (a) one at a time and (b) all at once to make the remaining network disconnected. We also checked for “*bottle-necks*” in the networks. We used the “min_cut” function of the “igraph” package of the R software [42].
A network is said to have a “*bottle-neck*” if there is a small set of edges in the network such that removal of the edges causes the network to split into two or more sizable, disconnected components [42].	

### Network Visualization

We used Gephi software to visualize the network sociograms for the four project periods [35]. Each stakeholder in the network is represented by a node, encoded to convey attributes of affiliation type (node shape), affiliated organization (node color), and leadership (node size). The arrangement of nodes in the sociogram is designed to aid visual comparisons between project periods. The weight of the edges between the nodes is visualized by line intensity and color on a green (less) to red (more) gradient. The interactions of individuals who are affiliated to more than one organization are represented only once in the creation of the network to reflect their primary affiliation.

### Individual-Level Interactions

An individual’s degree was computed using the notion of a weighted network. We used two-sample Wilcoxon tests to compare the distribution of degree among stakeholders across the four different project phases.

### Interactions between Role Groups

To characterize interactions over time between groups of stakeholders with different skills and perspectives, we aggregated the interactions between stakeholders by their roles and compared the interactions among role–role pairs across the project periods. We performed the Kolmogorov–Smirnov test [38] to compare interactions among role types.

### Centrality

To assess centrality, we used the “eigen_centrality” function of the R software, based on the method developed by Bonacich [41] because it incorporates the most measures of prestige or rank within the network.

### Vulnerability

We tested each of the four networks corresponding to project periods for vulnerability to random and purposeful loss of stakeholders.

## Results

### Network Size and Diversity

Forty-seven CAMP1/CAMP2 progress meetings were held across 89 months. The number of stakeholders participating in each project phase increased over time: 33 in CAMP1 Development, 46 in CAMP1 Implementation, 66 in CAMP2 Development, and 68 in CAMP2 Implementation.

In total, 141 stakeholders attended at least 1 CAMP1/2 progress meeting. Characteristics of the 82 (58%) who completed a demographics survey were age: 20% 18–34 years, 52% 35–54 years, 13% 55–64, 6% 65–74 years, and 9% age >75 years old; Sex: 57% female (including transgender female); Race: 25% Asian, 1% American Indian/Alaska Native, 24% Black or African-American, 48% White, and 8% other; and Ethnicity: 20% of Spanish, Latino/a or Hispanic descent. Ninety-two percent of respondents had attained more than 4 years of college education.

To simplify the analysis, stakeholders were sorted based on their protocol-related activities into seven functional project roles (Table 2A), and the affiliated organizations were sorted into six affiliation types (Table 2B). Individual stakeholders were further characterized as fulfilling leadership roles (e.g., medical, site, or core director, principal investigator, head of lab, etc.) or non-leadership roles in the project.

**Table 2.** Stakeholders’ institutional titles by project roles, organizations by type. (A) Stakeholder titles listed by project role (number of stakeholders)

**Administrator (Admin) (14)**	**Recruiter/Community Health Worker (Rec/CHW) (12)**
• Admin – Medical	• CHW – Trainer
• Admin – Research	• Community Health Worker (CHW)
• Program Officer	• Research Assistant
**Scientist (Sci) (9)**	• Recruiter
• Head of Laboratory (HOL)/Department Chair (3)	**Research Team-Other (RTO) (45)**
• Research Assistant (1)	• Commercial Partner – Collaborator
• Scientist – Early Career (2)	• Director of Research and Evaluation at Practice-Based Research Network (PBRN)*
• Scientist – Clinical Scholar (2)	• E-learning Staff
• Scientist – Other (1)	• Information Technology
**Clinician/Clinician Researcher (Clin) (36)**	• Intern
• Associate Medical Director*	• Medical Assistant
• Chief Medical Officer*	• Medical Student
• Chief of Clinical Strategy and Research*	• Program Director*
• Clinician – Doctor of Medicine (MD)/Nurse Practitioner (NP)/Physician Assistant (PA)	• Research Assistant
• Clinician – Nurse	• Project Manager
• Director of Research*	• Scientist – Social Network Analysis
• Medical Director*Medical Site Director of Internal Medicine*	• Scientist – Other
• Physician*	• Site Student
• Professor* (Infectious Diseases, Pharmacotherapy)	• Vice President for Clinical Affairs*
• Program Director – Translational Science Program*	**Rockefeller University Center for Clinical and Translational Science (RU-CCTS) (18)**
• Scientist – Clinical Scholar	• Administrative Director*
• Scientist – Early Career	• Biostatistician
• Scientist – Other	• Community Engagement Core Co-Director, Associate Professor*
• Vice President –Quality Improvement and Population Health*	• Community Engagement Core Co-Director, Professor *
**Community member (Comm) (7)**	• Community Engagement Specialist
• Grassroots community partner	• CTSA Principal Investigator (PI), Vice President, Professor, HOL, Scientist*
• Grassroots– patient	• Information Technology
• Volunteer	• President/Chief Executive Officer – PBRN • Scientist – Clinical Scholar, Other

* Leadership.

**Table 2B.** Organizations assigned to specific affiliation types in the Social Network Analysis*

**Academic (AC)**	**Community Partner (CP)**
• The Rockefeller University Center for Clinical and Translational Science	• Community Health Worker (CHW) Network of NYC
• University of California, Irvine	• Denny Moe’s Superstar Barbershop
• Washington State University	• Patient Stakeholder – Coney Island Hospital
• Weill Cornell Medical Center	• Patient Stakeholder – Lutheran Family Health Center
**Practice-Based Research Network (PBRN)**	• Patient Stakeholder – Metropolitan Hospital Center
• ACCESS Community Health Network – Chicago	**Funder (FND)**
• Clinical Directors Network (CDN)	• Agency for Healthcare Research and Quality (AHRQ)
• South Texas Ambulatory Research Network (STARnet) & The University of Texas at San Antonio	• National Institutes of Health (NIH)
**Federally Qualified Health Center/Community Health Center/Community Practice/Hospital (CHC)**	• Patient-Centered Outcomes Research Institute (PCORI)
• Brookdale Family Care Center	**Private Partners (PP)**
• Community Healthcare Network	• My Own Med (MOM)
• Coney Island Hospital	• VisualDx
• Hudson River Healthcare	
• Lincoln Hospital	
• Lutheran Family Health Centers	
• Lutheran Medical Center	
• Manhattan Physician Group/AdvantageCare Physician	
• Metropolitan Hospital Center	
• Open Door Family Medical Centers	
• Park Slope Family Health Center	
• Urban Health Plan	

* For some organizations, individual subsites are acknowledged here, whereas they are combined under one organization elsewhere in the manuscript. Thus, the total number of organizations may be slightly higher here.

#### Visualization of the Social Network, Individuals and Organization Types

**Fig. 1.**
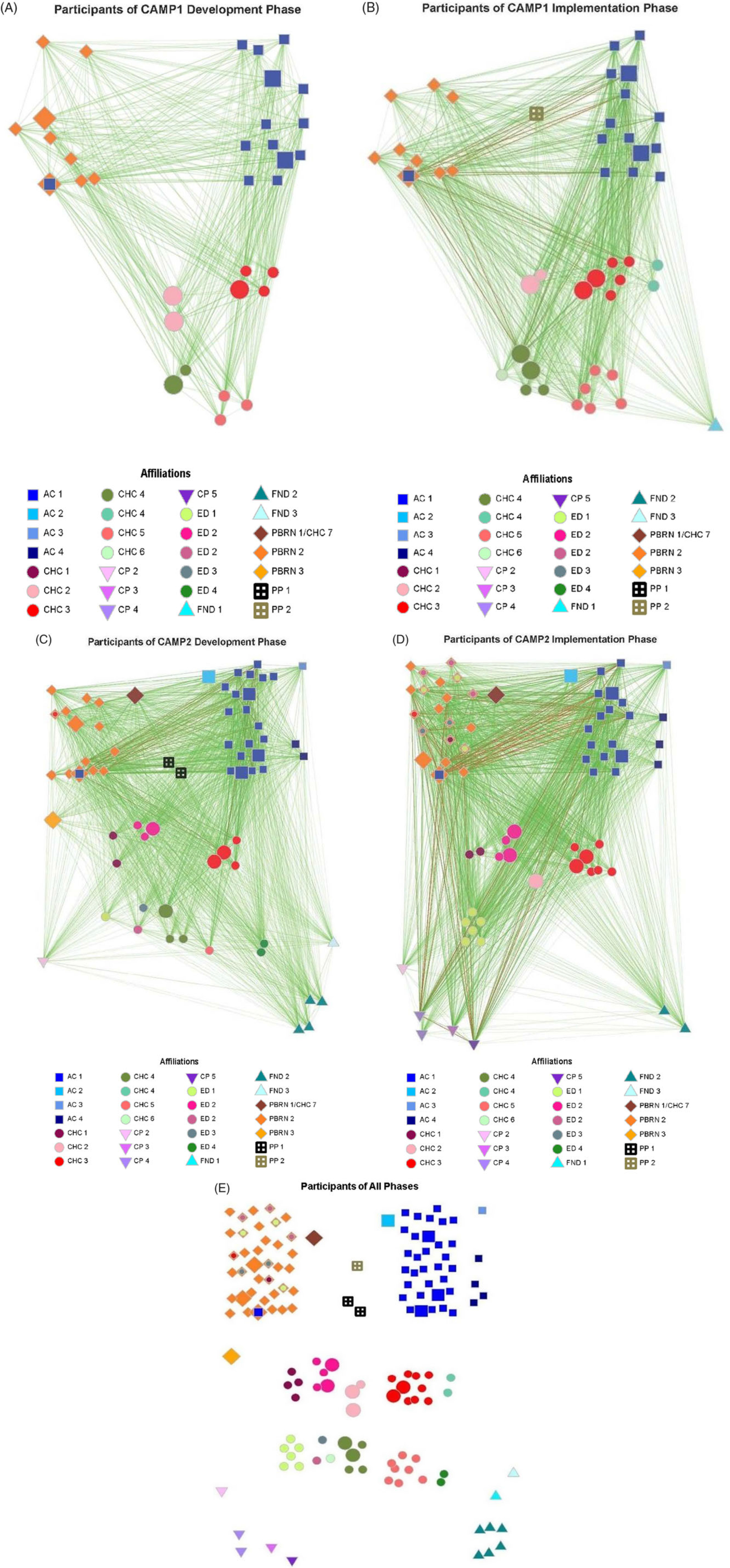
(A–E). Interactions among stakeholders according to their organization and affiliation type. Panels represent the social network for stakeholder interactions during CAMP1 Development (A), CAMP1 Implementation (B), CAMP2 Development (C), and CAMP2 Implementation (D). Panel E shows all stakeholders in the network. Each node represents an individual stakeholder. Shapes signify the organization types: 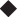 = Practice-Based-Research-Network (PBRN); 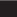 = Academic Institution (AC); 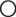 = Community Health Center (CHC); 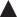 =Funder (FND); 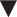 = Community Partner (CP); 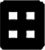 = Private Partner (PP)). The second colored shape inserted within a node indicates the second affiliation. The color of the node indicates the specific organization. Larger size nodes indicate stakeholders fulfilling leadership roles.

A network diagram was generated for stakeholder interactions in each of the four project phases. Over time, the networks grew larger and interactions among stakeholders increased (Fig. 1 A–E). The interactions across different organization types increased in successive project phases, with the greatest interactions among the stakeholders from the PBRNs and the academic institutions in the second and third phases, and among community partner affiliates, and the PBRNs in the last project phase.

#### Increasing Interactions at the Stakeholder Level

To look more closely at how stakeholders’ level of engagement changed across the project periods, we performed two-sample Wilcoxon tests to compare degree distributions of the stakeholders across the four different project phases. In the network from the first project phase, there was a higher density of stakeholders with a low degree representing little interaction with others in the network. As the project progressed, the number of stakeholders with very low degrees declined, and the level of stakeholder degree was more evenly distributed throughout the partnership. The change in the distribution of degree was significant from CAMP1 Development to CAMP1 Implementation (*P* = 0.014), and from CAMP1 Implementation to CAMP2 Development (*P* = 0.009). Stakeholder interactions overall did not further increase significantly in CAMP2 Implementation.

#### Interactions among Role Groups

Interactions increased significantly among stakeholders fulfilling different roles across the life of the project (Table 3). In the first project phase (CAMP1 Development, C1-D), there were too few time points to test for significant change. In the transition from the second to the third project phase, CAMP1 Implementation to CAMP2 Development, the interactions between Scientists (Sci) and Research Team-Other (RTO) increased significantly, as did those between the RU-CCTS CE core members and RTO stakeholders, with *p*-values 0.04 and 0.003, respectively. From the third project period (CAMP2 Development) to the last period (CAMP2 Implementation), interactions increased significantly between Community Members (patients) and every other group except administrators, and between the Recruiters/Community Health Workers and every other group except administrators (all *P* < 0.005).

**Table 3.** Interactions among partnership stakeholders* by role across project periods**

	RU-CCTS				Admin				Scientist				Clinician				RTO				Rec/CHW				Comm			
	C1-D	C1-I	C2-D	C2-I	C1-D	C1-I	C2-D	C2-I	C1-D	C1-I	C2-D	C2-I	C1-D	C1-I	C2-D	C2-I	C1-D	C1-I	C2-D	C2-I	C1-D	C1-I	C2-D	C2-I	C1-D	C1-I	C2-D	C2-I
RU-CCTS	28	76	180	159	0	5	49	45	23	134	250	186	85	255	263	270	65	171	**356**	**382**	0	0	17	**242**	0	4	8	**161**
Admin	0	5	49	45	0	0	6	11	0	3	22	18	0	5	49	37	0	5	51	48	0	0	2	32	0	0	0	7
Scientist	23	134	250	186	0	3	22	18	6	35	64	44	35	178	146	148	33	117	**203**	202	0	0	8	**112**	0	6	3	**82**
Clinician	85	255	263	270	0	5	49	37	35	178	146	148	48	157	90	87	88	209	251	292	0	0	13	**167**	0	6	2	**114**
RTO	65	171	**356**	**382**	0	5	51	48	33	117	**203**	202	88	209	251	292	31	61	152	171	0	0	22	**243**	0	8	5	**164**
Rec/CHW	0	0	17	**242**	0	0	2	32	0	0	8	**112**	0	0	13	**167**	0	0	22	**243**	0	0	1	**58**	0	0	0	**102**
Comm	0	4	8	**161**	0	0	0	7	0	6	3	**82**	0	6	2	**114**	0	8	5	**164**	0	0	0	**102**	0	1	0	25

* Stakeholder roles: RU-CCTS, The Rockefeller Center for Clinical and Translational Science; Admin, Administrator; Scientists; Clinicians, Clinician/Clinicians, Researcher; RTO, Research Team-Other; Rec/CHW, Recruiter/Community Health Worker; Comm, Community Member.

** Project phases: C1D, CAMP1 Development; C1I, CAMP1 Implementation; C2D, CAMP2 Development; C2I, CAMP2 Implementation.

Interactions: Cell values reflect the total number of interactions between members fulfilling roles in the intersecting column and row. Values are shaded from yellow (lowest) to green (highest) for ease in visual interpretation.

Bold type indicate a statistically significant change in the level of interaction compared to the prior project period.

#### Centrality

We calculated eigenvector centrality for all members of the network, and ranked stakeholders from highest to lowest eigenvector centrality for each project period. To compare the stakeholders most central to the networks in each period, the 15 stakeholders with the highest eigenvector centrality are shown by affiliation type and leadership status (Table 4). Individuals with leadership roles were prominent among the top 15 during the development phases of the 2 projects, CAMP1 (*n* = 6) and CAMP2 (*n* = 5), compared to the implementation phases (CAMP1 *n* = 5, CAMP2 *n* = 2). Seven of the 15 highest centrality stakeholders at the inception of the CAMP1/2 projects were from the CHCs, and 4 among them were members of CHC leadership. More stakeholders in non-leadership roles rose to the top 15 in centrality over the course of the 4 project periods (*n* = 9, 10, 10, 13, respectively). In the last project phase, in parallel with community members’ statistically significant increase in interactions with all other role types, the centrality of 3 community members/patients rose to be among the top 15. In addition, 3 CHC-based recruiter/CHW stakeholders, hired by the PBRN to support engagement at the CHC sites, also rose in centrality to rank among the top 15.

**Table 4.** Stakeholders with the Highest Eigen Centrality Scores* in the CAMP1/2 social network in each project period

	CAMP1 Development		CAMP1 Implementation		CAMP2 Development		CAMP2 Implementation	
Rank	Affiliation	Score	Affiliation	Score	Affiliation	Score	Affiliation	Score
1	PBRN/Academic – L*	1.00	PBRN	1.00	PBRN	1.00	PBRN/Academic – L*	1.00
2	Academic*	1.00	PBRN/Academic – L*	1.00	PBRN/Academic – L*	0.98	PBRN	0.97
3	PBRN	1.00	PBRN-L	0.88	Academic – L*	0.97	PBRN	0.90
4	CHC – L	1.00	Academic	0.88	Academic	0.95	Academic	0.88
5	CHC	1.00	Academic	0.82	PBRN	0.94	Academic*	0.78
6	CHC – L	1.00	Academic – L*	0.79	Academic	0.90	Academic*	0.76
7	Academic	0.83	CHC	0.77	Academic*	0.86	Community Partner	0.75
8	Academic – L*	0.83	Academic*	0.68	Academic	0.86	Community Partner	0.72
9	Academic – L*	0.83	Academic*	0.63	Academic*	0.84	CHC/PBRN	0.67
10	CHC	0.64	CHC	0.59	CHC – L	0.78	Community Partner	0.67
11	PBRN	0.45	CHC	0.54	Academic	0.78	PBRN	0.66
12	PBRN	0.45	CHC – L	0.52	Academic – L*	0.71	Academic – L*	0.66
13	CHC	0.45	CHC – L	0.47	Academic	0.60	Academic	0.62
14	CHC	0.45	Academic	0.45	CHC	0.56	CHC/PBRN	0.56
15	CHC – L	0.45	Academic	0.44	CHC – L	0.53	CHC/PBRN	0.55

Eigen centrality is scored between 0 and 1; values closer to 1 indicate higher centrality. Individual stakeholders are represented by their organization’s affiliation type, 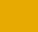 PBRN, Practice-Based-Research-Network; 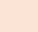 CHC, Community Health Center/Federally Qualified Health Center/Community Practice/Hospital; 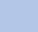 Academic; 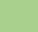
Community Partner. Additional designations are included for stakeholders with leadership roles at their institutions, (L) or with a role in the Community and Collaboration Core of the RU-CCTS (*).

#### Network Vulnerability

**Fig. 2.**
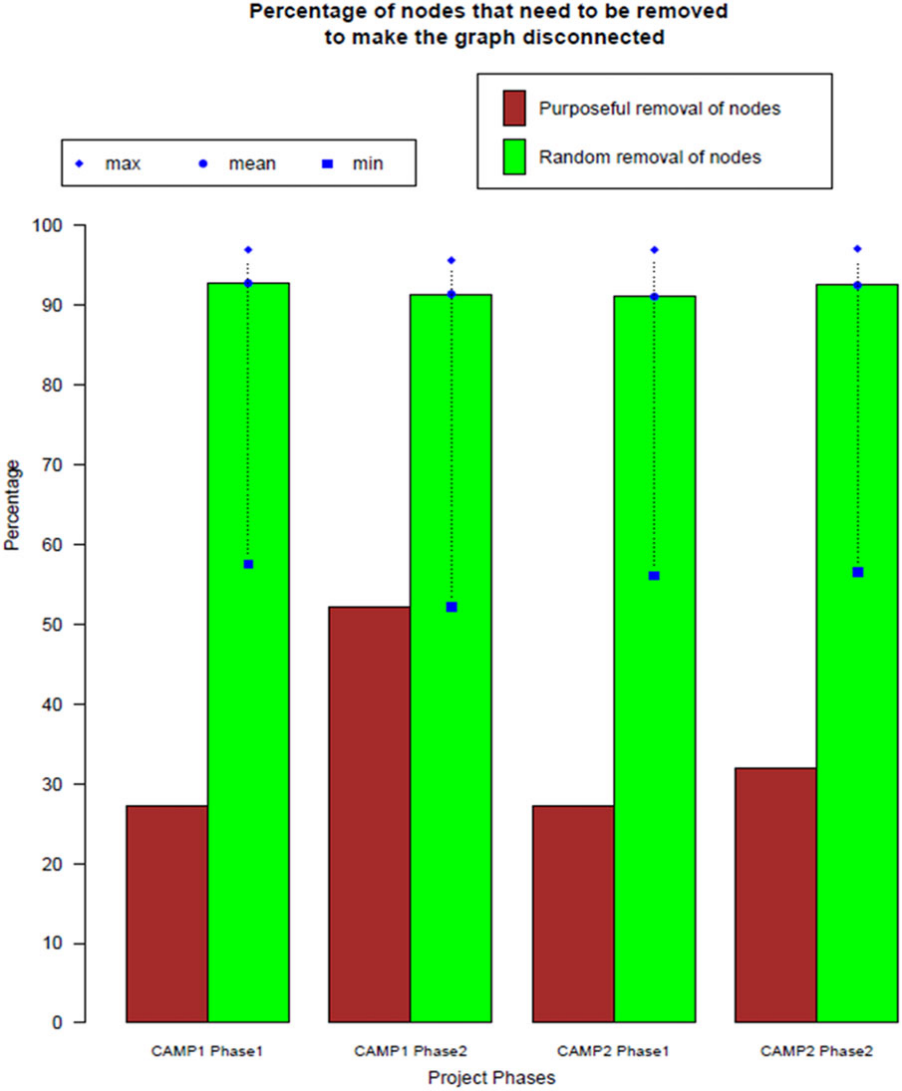
Vulnerability of the networks to loss of stakeholders in each project phase. The removal of stakeholders was modeled across the project phases using algorithms for random removal of stakeholders (green) or purposeful sequential removal of the network members with the highest degree in the network (red). The average percentage of stakeholders removed before the network fragmented is shown on the *y*-axis. Variance across 1000 replicates is shown.

The network was relatively resilient – able to withstand stakeholder loss without network fragmentation – in vulnerability analyses performed on each of the project phases (Fig. 2). Each of the four networks was minimally vulnerable to the random removal of up to 90% of stakeholders. Networks were more vulnerable to targeted damage. The implementation phase of CAMP1 was the least vulnerable to targeted damage – 50% of highest degree stakeholders could be removed before the network fragmented; in comparison, the development phase of CAMP1 and both phases of CAMP2 were each damaged upon the loss of 28%–30% of the highest degree stakeholders.

#### Associations of Social Network Measures with Study Milestones

**Fig. 3.**
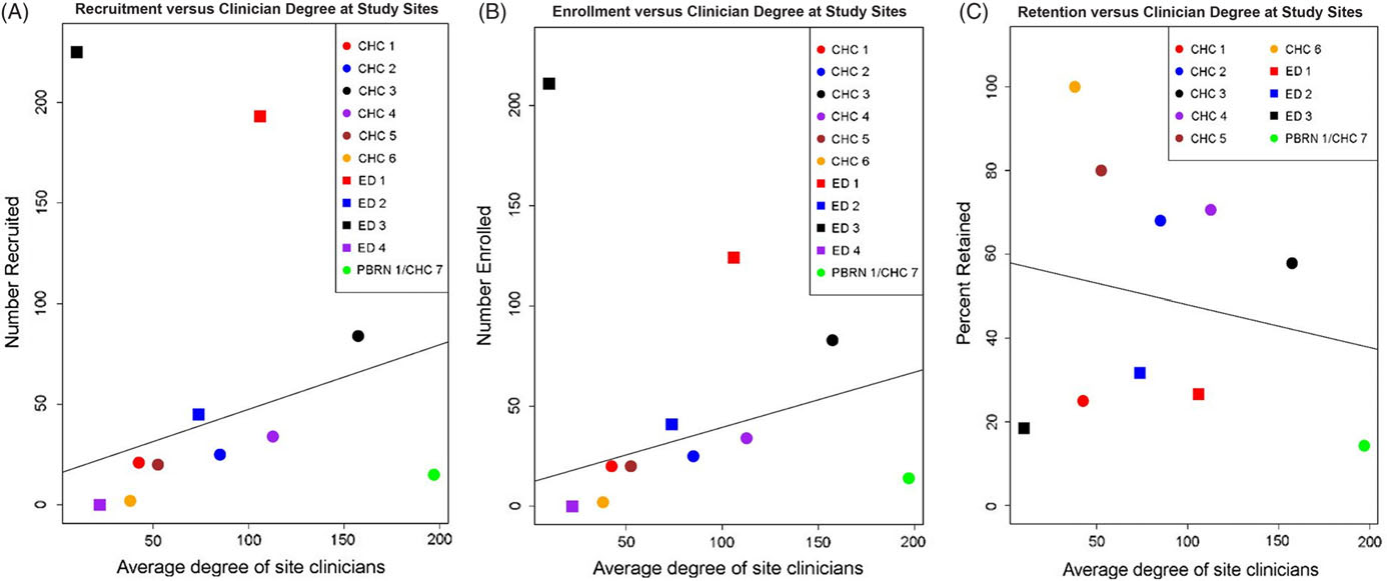
(A–C). Association of CAMP1 and CAMP2 study milestones with clinician engagement in the network. The number of participants recruited (A), enrolled (B), and retained through all study visits (C) is plotted against the average degree in the network of the clinicians affiliated with the site. Sites are Community Health Centers (CHCs), Federally Qualified Health Centers and Community Practices, a Practice-Based Research Network (PBRN) contributing two CHC sites (PBRN/CHC), and Emergency Departments (ED).

We hypothesized that the level of engagement of site clinicians in the partnership might be positively associated with their sites’ success in recruitment, enrollment, and retention. To test for association, we plotted the average degree of a site’s clinicians in the network, against the number of participants recruited, enrolled, and retained at that site (Fig. 3 A-C). Overall, site recruitment and enrollment were positively associated with average clinician stakeholder degree in the network. The two sites that were outliers for recruitment and enrollment, ED1 and ED3, had extremely proficient recruiters. Retention was not associated with clinician degree in the partnership network.

## Discussion

The CAMP1/CAMP2 collaborators formed a successful, sustainable community–academic research partnership that developed and completed two large extramurally funded CEnR/CER research projects translating observations from basic and clinical sciences into practice-based, and home-based interventions. The partnership employed principles of CBPR and PCORI rubrics [15] and the operational models of the partnering PBRN and CEnR-Nav [13]. We used attendance at progress meetings to define interactions in our SNA to explore the evolution of interactions in the partnership over time, based on the rationale that meetings were highly interactive and participatory by design, and brought together individuals that might not have interacted directly otherwise during the project. To add rigor to this simple interaction measure, we used the notion of a weighted network when calculating stakeholder’s degree in the network and selected a measure of centrality that incorporates the notion of rank in the network to assess the relative importance of each stakeholder. The social networks formed by the CAMP1/CAMP2 partnership grew in size, degree, and complexity over the life of the projects, with increasing connectedness among organization types, partnership roles, and individuals over time. Specifically, the increasing interactions of the Community Members with other role groups, and of the Recruiter/Community Health Workers with almost all other groups increased significantly during the fourth project period reflecting the realization of the design and aims of the project to assure engagement of communities, patients, and diverse stakeholders together in research across the life of the project. The rise in the centrality of Community Members through the CAMP2 project to be among the top 15 for centrality is another tangible measure of participation in the project partnership. The limited interactions with administrators may be attributable to their specific roles as agency officials who visited intermittently and administrators with roles in support of grant writing and study operations.

The interaction maps demonstrate the explicit intention of RU-CCTS/CDN to foster engagement of community members and scientists early in the design and execution of translational research to create full spectrum translational research teams. The interactions among academic/scientists and community/clinicians were sustained from beginning to end of the CAMP1/CAMP2 projects. The successful outcomes of this ongoing engagement is evident in publications across the translational spectrum illuminating molecular findings [20,21], clinical observations [19,23,25], and aspects of implementation [13,22,24]. In CAMP1, the Community Clinicians from CHCs, many of whom fulfill leadership roles at their sites, were brought to the partnership through their relationship to the CDN PBRN and formed the initial critical bridge between the community and the scientists in this network. In CAMP2, the networks involved patients as the direct representatives of the community, as well as community health workers and nonacademic partners, all strengthening the bridges between community and academic partners, and realizing RU-CCTS/CDN and PCORI principles of engagement. Patients and community advocates who have served as stakeholders for research often have compelling stories that amplify the impact of the data. Their interactions with all members of the partnership have implications for the translation of the work into policy and practice in the healthcare settings where the study took place, and for enhancement of the partnership engagement process into other translational CEnR endeavors.

This SNA of the CAMP partnerships adds to the evidence base demonstrating the network characteristics resulting from an effective approach to building effective community–academic partnerships. The ongoing and increasing engagement across different members of the network implies effective partnership building, and the growth of trust, shared values, and purpose. The SNA characterizes a successful partnership network for research, which simultaneously addresses basic science questions of T1/T2 early phase translational research [18] within the context of clinical effectiveness studies of later translational phases (T3/T4) while examining outcomes that matter to both clinicians and patients.

Vulnerability analysis revealed the partnership network to be resilient. The apparent resistance of the network to random stakeholder loss reflects a strength of centralized progress meetings that sustain a connection to all stakeholders, as demonstrated by the absence of a bottleneck. Some level of redundancy within the partnership such as engagement of many CHC sites and overlapping expertise from multiple institutions may have added to resilience.

There are several limitations to the study. We inferred interactions from attendance at regularly scheduled progress meetings that employed a collaborative participatory model [23]. The network analysis did not account for interactions that occurred outside of progress meetings that may have contributed to network cohesion or to the creation of subnetworks we could not detect using this approach. It might have enriched the analysis to have collected qualitative data or validated assessments from the stakeholders specifically addressing their engagement experiences and perceived partnership strengths and weaknesses [5]. During CAMP1 planning and conduct, we discussed collaboration assessment tools with the stakeholders and distributed an assessment tool, which stakeholders uniformly did not complete. In a subsequent discussion, partners indicated they were eager to collaborate, but preferred not to be studied.

This SNA was conducted retrospectively. Lessons learned that might be applied prospectively to an evolving partnership include (1) data routinely collected in the course of project operations – such as attendance data, meeting notes, stakeholder characteristics, and study milestones – can provide a rich source of information about the partnership to complement or guide selective use of more labor-intensive qualitative assessments. Prospective planning and the use of structured tools for routine data capture improve data quality; (2) gaining stakeholder enthusiasm for the use of partnership assessment measures is important and could extend and validate network insights; (3) a variety of analysis tools can be used to reveal different features of a network, such as patterns of interactions or critical bridges among network components; (4) centrality analyses can be helpful to identify stakeholders who may be facing barriers to full participation in the network; (5) assessing network vulnerability during an evolving partnership could improve network cohesion. Network relationships that form bridges across organization types, such as the dual affiliations of PBRN/Academic partners, the role of PBRNs in facilitating research at CHCs, or the strategic embedding of PBRN/CHC stakeholders as recruiters, can help to keep the network connected.

The social network of the CAMP1/2 research partnership grew and gained complexity through the life of two major externally funded projects spanning 8 years. SNA analysis afforded insights into the robustness of the network and revealed the course of specific group-group interactions over time. Scientists, RU-CCTS leadership, clinicians, and CDN-PBRN members were engaged early in the study development and conduct of CAMP1. The interactions of community partners with most other stakeholder groups increased significantly during the implementation of CAMP2, and community partners rose to have high network centrality by study completion. SNA provided tangible evidence of realization of NCATS, RU-CCTS, and PCORI principles of engagement. Lessons from this SNA could be applied to other partnerships midcourse to gain valuable insights.
